# Gene expression analysis of colon high-grade dysplasia revealed new molecular mechanism of disease 

**Published:** 2018

**Authors:** Habib Malekpour, Mohammad Hossein Heidari, Reza Vafaee, Hamideh Moravvej Farshi, Mahsa Khodadoostan

**Affiliations:** 1 *Gastroenterology and Liver Diseases Research Center, Research Institute for Gastroenterology and Liver Diseases, Shahid Beheshti University of Medical Sciences, Tehran, Iran*; 2 *Student Research Committee, Proteomics Research Center, Faculty of Paramedical Sciences, Shahid Beheshti University of Medical Sciences, Tehran, Iran*; 3 *Proteomics Research Center, Shahid Beheshti University of Medical Sciences, Tehran, Iran*; 4 *Skin Research Center, Shahid Beheshti University of Medical Sciences, Tehran, Iran*; 5 *Department of Gastroenterology and Hepatology, Faculty of Medicine, Isfahan University of Medical Sciences, Isfahan, Iran*

**Keywords:** Transcriptome, Interactome, Colon cancer, High grade dysplasia

## Abstract

**Aim:**

The aim of this research was to find a clear molecular view of dysplasia via network analysis.

**Background:**

There are some evidence suggest the relationship between dysplasia and colorectal cancer. Understanding of high-grade dysplasia (HGD) could be beneficial for colon cancer management.

**Methods:**

Bioinformatics study of HGD versus healthy subjects was conducted to check the status of differentially expressed genes (DEGs). GSE31106, GPL1261, GSM770092-94 and GSM770101-6 were the sources from gene expression omnibus (GEO) that queried for protein-protein interaction (PPI) network analysis via Cytoscape and its algorithms. Hubs of network were enriched for biochemical pathways and were validated via clustering analysis.

**Results:**

Numbers of 46 hub nodes were determined and were included in 12 pathways. A main cluster including 76 nodes was identified containing 45 hubs. 33 hubs among 46 genes were involved in biochemical pathways. IL1B, IL6, TNF, and TRL4 were the most important critical genes.

**Conclusion:**

Many different genes as hub nodes might influence the trigger and development of advance condition and also colon cancer.

## Introduction

 Colon cancer accounts for the third cause of cancer death in the world (1). A need for early detection and management of this malignant tumor is much sensed. In this regard, pre-modifications known as precancerous conditions could be important to study for introduction of molecular targets (1, 2). High-grade dysplasia (HGD) refers to a condition prior to cancer occurrence. High throughput molecular analysis of this condition is a way to get a better knowledge of both dysplasia condition and its progressive form known as colon cancer (1, 3). Sequential modifications occur in the genetic basis and cancers such as colorectal manifests. Oncogenes and tumor suppressor genes have a role in these changes with opposite behaviors. The series of alteration is normally from benign adenomas to colorectal carcinomas and in its way, it modifies from low-grade dysplasia to HGD. Accumulating abnormal gene expressions such as APC, p53, DCC, survivin and RAS have a link with this transition as the normal tissue develops precancerous and eventually cancerous stages (4). Therefore, patients with dysplastic lesions are important to be investigate d for follow-up and screening in case of cancer developments. What is more about these molecular targets at the precancerous conditions, is that to find the most accurate and specific ones (2). These agents could be investigated for topological features in a whole interaction map. In this way, more support and validation can be achieved. These maps are present in organisms as a protein-protein interaction network via physical correlations (5). Functions carried out in a cell is due to the normal interactions of these molecules. When it comes to the interaction pattern of molecules such as genes and proteins, normal expression and their functions are the key. Changes in expression of any of these genes, microRNAs, and proteins could result in abnormal interactions (6). The worst part is when these genes are with high centrality and are encountered with expression changes. Furthermore, gene ontology analysis of the central elements could provide more information about the disease underlying mechanisms (7). It can show which related biological process to these central agents could be disrupted and therefore worth more evaluations. To reach this goal, there are algorithms available that could analyze the genes corresponding terms. In this research, protein-protein interaction network of differentially expressed genes of HGD in comparison with normal condition is analyzed to introduce the most promising ones for clinical applications. 

## Methods

**Data collection**

Microarray Data (GSE31106, GPL1261, GSM770092-94 and GSM770101-6) were considered to compare Gene expression profiles of normal group and treated five-week-old male mice with intraperitoneal injected with 10mg/kg Azoxymethane. The samples three cycles with Dextran sulfate sodium (2%, 1.5%, and 1.5%) were treated while controls were treated with salin injection and drinking distilled water. The extracted RNAs from colorectal tissue after 6 weeks of treatment were analyzed by Affymerix GeneChip Mouse Genome 430 2.0 Array. Characterized DEGs with fold change (FC) less than 0.5 and more than 2 among the 250 top significant DEGs were selected to be examination via PPI network analysis.

**PPI network analysis**

STRING database (8) and Cytoscape software (9) version 6.3.2 were applied to construct PPI network. The network was evaluated by Network Analyzer application of Cytoscape and the hubs were selected based on degree cutoff (mean+2SD) (10). Related biological processes to the hub nodes were identified by ClueGO v 2.5.0 plugin of Cytoscape software from KEGG 20.11.2017. 

**Statistical analysis**

Gene expression profiles were matched via boxplot analysis and p-value <0.05 was considered for significant findings. Biological processes were identified based on K-score, at least 10 genes/Term, and 10% participation of genes per term. 

## Results

**Figure 1:**
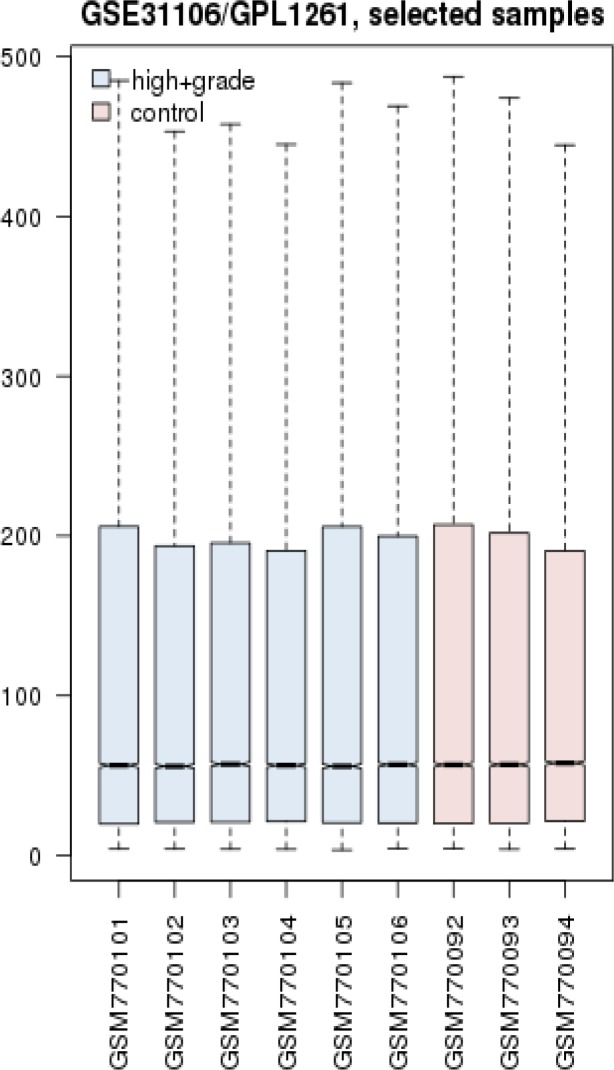
Gene expression profiles of 6 HGD mice and 3 normal controls

**Figure 2:**
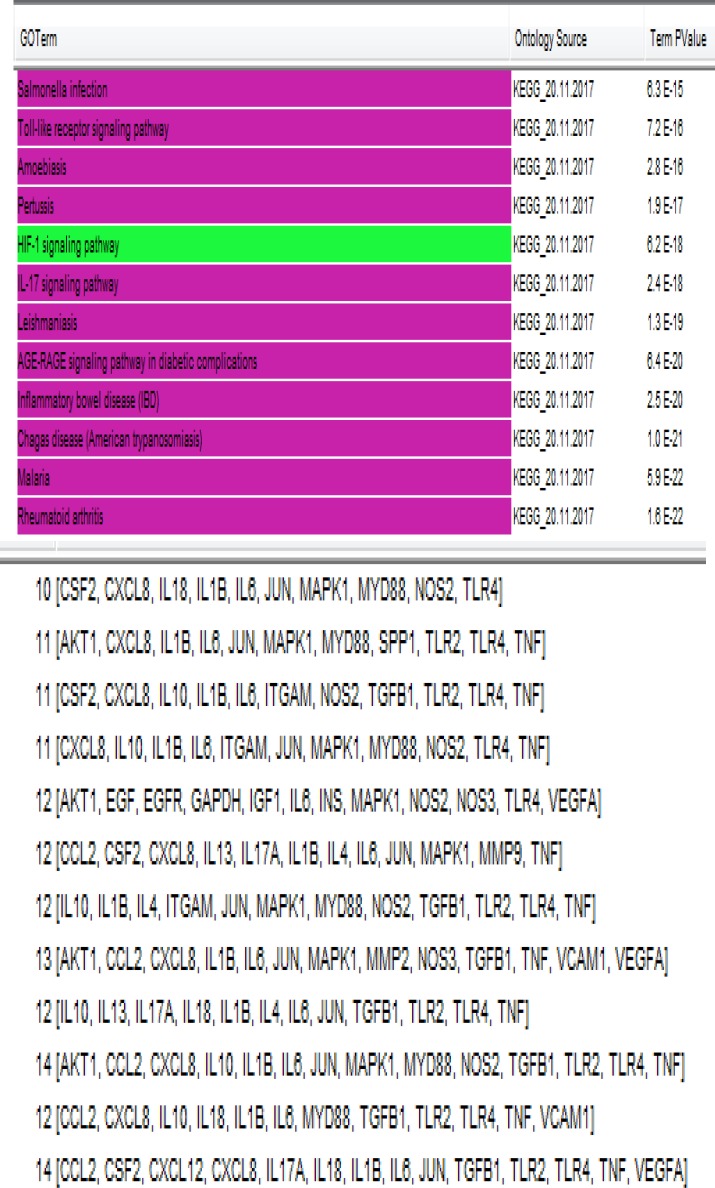
Pathways related to the hubs of dysplasia network. The involved genes in each process are presented in the bottom of figure. Except the green colored one, the other pathways are grouped in one cluster. Sequence of genes rows is corresponded with pathways up to down, respectively

**Figure 3:**
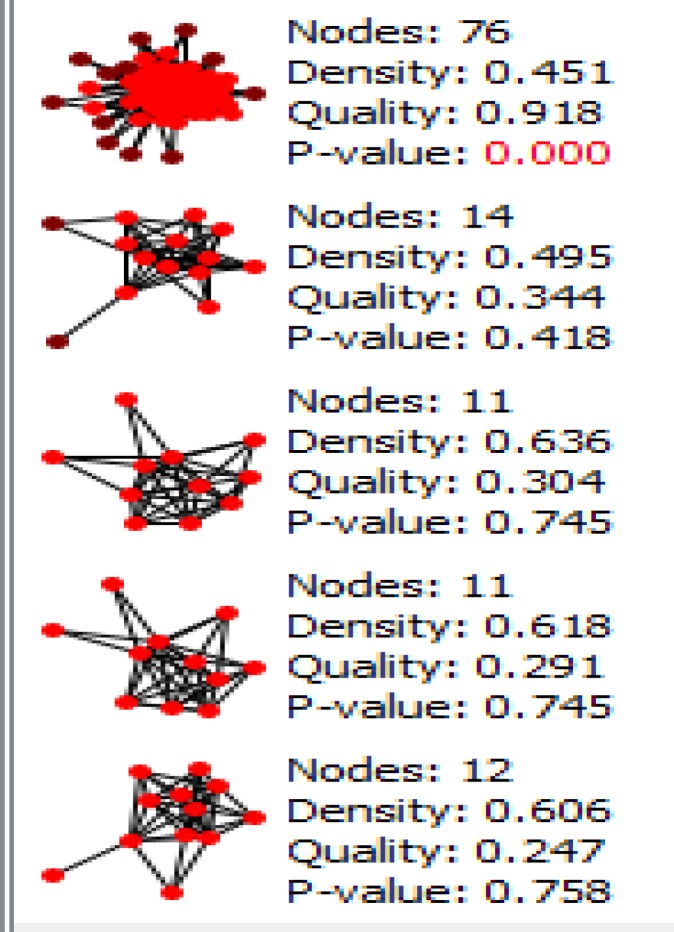
Protein cluster analysis of hub nodes via ClusterOne application is shown. The cluster was elected based on at least ten genes per cluster

**Figure 4:**
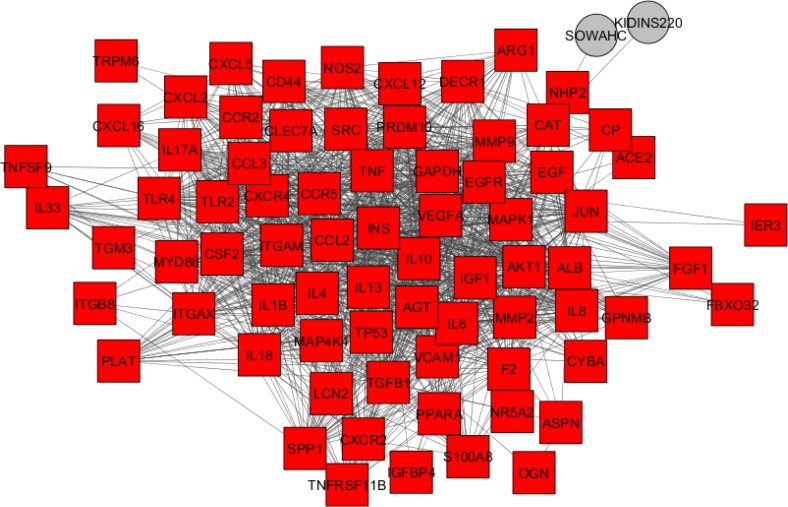
Cluster 1 PPI network. The two out layer nodes are displayed in gray color

Gene expression profiles of 6 HGD samples and 3 controls were matched via box plot analysis. As it is shown in the figure 1, the samples are comparable. It is appeared that 50% of genes are characterized in high levels of expression in both HGD and normal control samples. Numbers of 250 top significant DEGs based on p-value criterion were selected. Among 250 selected DEGs numbers of 24 individual were not characterized which excluded for more analysis. Numbers of 129 DEGs amongst 226 significant and characterized DEGs were included in PPI network analysis based on fold change less than 0.5 and above 2. STRING database recognized 98 DEGs and network was constructed via these ones and 50 added relevant genes. The network was included 34 isolated nodes and a main connected component. This component which we call it as network of HGD, contains 114 nodes and 1451 edges. Centrality analysis leds to introduce 46 hub nodes that are tabulated in the table 1. Betweenness centrality (BC) and closeness centrality (11) of the determined hubs are presented in this table. Numbers of 12 pathways related to the 46 hub nodes from KEGG were identified (Figure 2). As it is shown in figure 2, 32 hubs are involved in these pathways. Two hub nodes; IL1B and IL6 are complicated in 92% of pathways while EGF, EGFR, GAPDH, IGF1, INS, MMP2, MMP9, CXCL12, SPP1 are convoluted in one term, see table 2. The network was analyzed for protein clustering by ClusterOne plug-in, Cytoscape as indicated in figures 3 and 4.

**Table 1:** Hub nodes of HGD network

R	display name	Description	Degree	BC	CC
1	GAPDH	glyceraldehyde-3-phosphate dehydrogenase	67	0.059	0.706
2	IL6	interleukin 6 (interferon, beta 2)	64	0.021	0.677
3	TNF	tumor necrosis factor	63	0.025	0.673
4	INS	Insulin	63	0.058	0.681
5	TGFB1	transforming growth factor, beta 1	58	0.071	0.657
6	IL8	interleukin 8	58	0.026	0.653
7	ALB	Albumin	58	0.019	0.653
8	CCL2	chemokine (C-C motif) ligand 2	58	0.012	0.657
9	AKT1	v-akt murine thymoma viral oncogene homolog 1	58	0.046	0.661
10	VEGFA	vascular endothelial growth factor A	57	0.029	0.649
11	IL1B	interleukin 1, beta	56	0.024	0.646
12	JUN	jun proto-oncogene	56	0.021	0.661
13	PRDM10	PR domain containing 10	56	0.027	0.657
14	IL10	interleukin 10	54	0.009	0.638
15	IL4	interleukin 4	54	0.013	0.638
16	CSF2	colony stimulating factor 2 (granulocyte-macrophage)	54	0.012	0.638
17	MMP9	matrix metallopeptidase 9 (gelatinase B, 92kDa gelatinase, 92kDa type IV collagenase)	53	0.005	0.631
18	TLR4	toll-like receptor 4	52	0.008	0.631
19	DECR1	2,4-dienoyl CoA reductase 1, mitochondrial	51	0.046	0.635
20	SRC	v-src sarcoma (Schmidt-Ruppin A-2) viral oncogene homolog (avian)	51	0.015	0.631
21	VCAM1	vascular cell adhesion molecule 1	51	0.009	0.628
22	NOS3	nitric oxide synthase 3 (endothelial cell)	50	0.028	0.628
23	TP53	tumor protein p53	49	0.051	0.632
24	CXCR4	chemokine (C-X-C motif) receptor 4	48	0.007	0.608
25	ITGAM	integrin, alpha M (complement component 3 receptor 3 subunit)	48	0.011	0.611
26	IL13	interleukin 13	48	0.008	0.611
27	IL17A	interleukin 17A	47	0.025	0.595
28	TLR2	toll-like receptor 2	47	0.014	0.604
29	MAPK1	mitogen-activated protein kinase 1	47	0.019	0.614
30	EGFR	epidermal growth factor receptor	46	0.047	0.604
31	F2	coagulation factor II (thrombin)	45	0.015	0.608
32	EGF	epidermal growth factor	45	0.012	0.604
33	CXCL12	chemokine (C-X-C motif) ligand 12	45	0.003	0.592
34	CD44	CD44 molecule (Indian blood group)	44	0.004	0.598
35	IL18	interleukin 18 (interferon-gamma-inducing factor)	43	0.003	0.579
36	CAT	Catalase	43	0.040	0.601
37	IGF1	insulin-like growth factor 1 (somatomedin C)	43	0.007	0.592
38	NOS2	nitric oxide synthase 2, inducible	43	0.014	0.598
39	SPP1	secreted phosphoprotein 1	41	0.021	0.577
40	MMP2	matrix metallopeptidase 2 (gelatinase A, 72kDa gelatinase, 72kDa type IV collagenase)	41	0.002	0.582
41	ITGAX	integrin, alpha X (complement component 3 receptor 4 subunit)	40	0.006	0.562
42	MYD88	myeloid differentiation primary response 88	39	0.032	0.562
43	CCR5	chemokine (C-C motif) receptor 5 (gene/pseudogene)	37	0.002	0.554
44	AGT	angiotensinogen (serpin peptidase inhibitor, clade A, member 8)	36	0.008	0.559
45	CCR2	chemokine (C-C motif) receptor 2	35	0.001	0.549
46	CXCR2	chemokine (C-X-C motif) receptor 2	34	0.001	0.538

## Discussion

HGD is important to study for colorectal cancer management. This precancerous stage could be essential for identification of factor triggering tumorigenesis. One way to reach this purpose is through molecular research. Data mining; in addition, could assist adding more information and values related to the identified molecular agents corresponding to any conditions (12). Protein-protein interaction network is one of assessment biomarkers in terms of centrality role in an interaction network. In this network analysis approach, we identify central of differentially expressed genes network in HGD via associated methods and algorithms. To do this, at first, the quality of expression profile of samples of healthy and dysplasia groups were compared in figure 1. The analysis shows that the data is suitable for comparison as the samples are median-centered. 

GEO2R identified genes with modified expressions and these genes were queried in Cytoscape for a network construction. The network centrality analysis introduced 46 hub genes that almost none were from DEGs of HGD. Moreover, these genes are very close in degree values and could be very important in the network integrity. Among 114 nodes, 46 individuals were identified as hubs. On the other hand, about 40% of nodes are hubs. What is more, these genes are divided in four categories consisted of immune related genes (such as ILs), oncogenes (as like AKT1, JUN, and SRC), metabolism related genes (especially INS), and other types of genes (13-15). 

**Table 2:** Hub nodes that are involved in the 12 pathways

R	Gene name	Number of relevant pathways
1	IL1B, IL6	11
2	TNF, TRL4	10
3	JUN	9
4	CXCL8, MPK1	8
5	TLR2, TGFB1	7
6	NOS2, MYD88, IL10	6
7	CCL2	5
8	CSF2, IL18, AKT1	4
9	ITGAM, VEGFA, IL17A, IL4	3
10	NOS3, VCAM1, IL13	2
11	EGF, EGFR, GAPDH, IGF1, INS, MMP2, MMP9, CXCL12, SPP1	1

Oncogenes including AKT1, JUN, SRC are a gene set that are also prominent in colon cancer (16). The other genes belong to metabolic pathways are GAPDH, INS, IGF1 which play significant role in proliferation and apoptosis in colon cancer (11, 17). 

Functional categorization of hub genes indicated 12 associated pathways in figure 2 that except one of the pathway, HIf-1 signaling pathway which highlighted in green color, other terms are presented in the same group. Distribution of hub genes in the related pathways was analyzed in table 2. The findings indicate that IL1B and IL6 are mostly involved in the all biological terms similarly TNF and TRL4 as next rank participate in 10 biological terms. These elements of these two rows are all linked to immune system category. 

Furthermore, clustering analysis leds to introduce one significant cluster which contains approximately all the hubs except for NOS3. This clustering could validate the importance of these identified hubs in the HGD network. To get a better understanding, a literature review of the hub genes that are present in the most pathways (the first two rows) as well as the first two top ones among 46 genes is conducted for possible relationship with colon cancer. GAPDH, glyceraldehyde-3-phosphate dehydrogenase as a pleiotropic enzyme (18) has the highest degree, betweenness, and closeness amounts in this network and it is active in apoptosis (19). This gene has been referred with highlighted moonlighting effect in cancer development (20). It shows it is a possible key role in transition from dysplasia to cancer states. In addition, it’s up-regulation has been reported for colorectal cancer (17). IL 6 the next gene that is very important in cancer, its increment has been also associated with colorectal cancer progression (21). The higher the level of IL 6 in human serum, the more developed the tumor (22). This gene is also ranked as the first group in table 2. In this grouping, IL1B as another inflammatory system gene that is famous in gastrointestinal system and promotes invasion in colorectal tumor as well (23). TNF in the next group, high singling levels could be important in colon cancer (13). TRL4 is also reported for colon metastasis. In fact, multiple roles has been identified for this gene (24). 

In this research, it was confirmed that advance dysplasia is accompanied with vast alterations in gene expression algorithm of human body. In this regard immune system, metabolic pathways, and oncogenes are affected. In addition, deregulation of immune system and inflammation is prominent in HGD. This complexe condition in HGD may led to onset of colorectal cancer (25).

A further comprehensive knowledge of colorectal cancer and its prediction are interpreted by identification of crucial genes, which are involved in HGD (26, 27). This set of possible biomarkers and the related biological processes may play critical roles in transition between HGD and colon cancer. However, the exact participation of these genes will require more in-depth research for clinical setting.

## Conflict of interests

The authors declare that they have no conflict of interest.

## References

[1] Hartmans E, Orian-Rousseau V, Matzke-Ogi A, Karrenbeld A, de Groot DJA, de Jong S (2017). Functional genomic mRNA profiling of colorectal adenomas: identification and in vivo validation of CD44 and splice variant CD44v6 as molecular imaging targets. Theranostics.

[2] Bashizadeh-Fakhar H, Rezaie-Tavirani M, Zali H, Faraji R, Nejad EK, Aghazadeh M (2018). The Diagnostic Value of Serum CEA, CA-125, and ROMA Index in Low-Grade Serous Ovarian Cancer. Int J Cancer Manag.

[3] Larki P, Gharib E, Yaghoob Taleghani M, Khorshidi F, Nazemalhosseini-Mojarad E, Asadzadeh Aghdaei H (2017). Coexistence of KRAS and BRAF Mutations in Colorectal Cancer: A Case Report Supporting The Concept of Tumoral Heterogeneity. Cell J.

[4] Sharifian A, Pourhoseingholi MA, Emadedin M, Rostami Nejad M, Ashtari S, Hajizadeh N (2015). Burden of Breast Cancer in Iranian Women is Increasing. Asian Pac J Cancer Prev.

[5] Mansouri V, Zadeh-Esmaeel M-M, Rostami-Nejad M, Rezaei–Tavirani M (2018). Comparative study of gastric cancer and chronic gastritis via network analysis. Gastroenterol Hepatol Bed Bench.

[6] Koushki M, Atan NAD, Omidi-Ardali H, Tavirani MR (2018). Assessment of Correlation Between miR-210 Expression and Pre-Eclampsia Risk: A Meta-Analysis. Rep Biochem Mol Biol.

[7] Rezaei–Tavirani M, Tavirani SR, Rostami FT (2018). Biochemical pathway analysis of gastric atrophy. Gastroenterol Hepatol Bed Bench.

[8] Eesteghamati A, Gouya M, Keshtkar A, Najafi L, Zali MR, Sanaei M (2009). Sentinel hospital-based surveillance of rotavirus diarrhea in iran. J Infect Dis.

[9] Zhi J, Sun J, Wang Z, Ding W (2018). Support vector machine classifier for prediction of the metastasis of colorectal cancer. Int J Mol Med.

[10] Safari-Alighiarloo N, Rezaei-Tavirani M, Taghizadeh M, Tabatabaei SM, Namaki S (2016). Network-based analysis of differentially expressed genes in cerebrospinal fluid (CSF) and blood reveals new candidate genes for multiple sclerosis. Peer J.

[11] Nazemalhosseini Mojarad E, Kuppen PJ, Aghdaei HA, Zali MR (201). The CpG island methylator phenotype (CIMP) in colorectal cancer. Gastroenterol Hepatol Bed Bench.

[12] Azodi MZ, Rezaei-Tavirani M, Nejad MR, Rezaei-Tavirani M (2018). Human Prolactinoma: A View of Protein-Protein Interaction Pattern. Int J Endocrinol Metab.

[13] Kraus S, Arber N (2009). Inflammation and colorectal cancer. Current opinion in pharmacology.

[14] Watt SK, Hasselbalch HC, Skov V, Kjær L, Thomassen M, Kruse TA (2018). Whole Blood Gene Expression Profiling in patients undergoing colon cancer surgery identifies differential expression of genes involved in immune surveillance, inflammation and carcinogenesis. Surg Oncol.

[15] Principe DR, DeCant B, Mangan RJ, Wayne EA, Diaz AM, Vitello D (2015). TGFβ signaling deficiency enhances tumor associated inflammation in colon cancer. AACR.

[16] Rostami Nejad M, Rostami K, Cheraghipour K, Nazemalhosseini Mojarad E, Volta U, Al Dulaimi D (2011). Celiac disease increases the risk of Toxoplasma gondii infection in a large cohort of pregnant women. Am J Gastroenterol.

[17] Tang Z, Yuan S, Hu Y, Zhang H, Wu W, Zeng Z (2012). Over-expression of GAPDH in human colorectal carcinoma as a preferred target of 3-bromopyruvate propyl ester. J Bioenerg Biomembr.

[18] Tarze A, Deniaud A, Le Bras M, Maillier E, Mollé D, Larochette N (2007). GAPDH, a novel regulator of the pro-apoptotic mitochondrial membrane permeabilization. Oncogene.

[19] Sirover MA (2017). GAPDH: A Multifunctional Moonlighting Protein in Eukaryotes and Prokaryotes Moonlighting Proteins. Novel Virulence Factors in Bacterial Infections.

[20] Sirover MA (2018). Pleiotropic effects of moonlighting glyceraldehyde-3-phosphate dehydrogenase (GAPDH) in cancer progression, invasiveness, and metastases. Cancer Metastasis Rev.

[21] Tajbakhsh M, García Migura L, Rahbar M, Svendsen CA, Mohammadzadeh M (2012). Antimicrobial-resistant Shigella infections from Iran: an overlooked problem?. J Antimicrob Chemother.

[22] Knüpfer H, Preiss R (2010). Serum interleukin-6 levels in colorectal cancer patients—a summary of published results. Int J Colorectal Dis.

[23] Li Y, Wang L, Pappan L, Galliher-Beckley A, Shi J (2012). IL-1β promotes stemness and invasiveness of colon cancer cells through Zeb1 activation. Molecular cancer.

[24] Yesudhas D, Gosu V, Anwar MA, Choi S (2014). Multiple roles of toll-like receptor 4 in colorectal cancer. Front Immunol.

[25] Nazemalhosseini Mojarad E, Farahani RK, Haghighi MM, Aghdaei HA, Kuppen PJ, Zali MR (2013). Clinical implications of BRAF mutation test in colorectal cancer. Gastroenterol Hepatol Bed Bench.

[26] Norouzinia M, Asadzadeh H, Shalmani HM, Al Dulaimi D, Zali MR (2012). Clinical and histological indicators of proximal and distal gastric cancer in eight provinces of Iran. Asian Pac J Cancer Prev.

[27] Zamanian Azodi M, Peyvandi H, Rostami-Nejad M, Safaei A, Rostami K, Vafaee Ret al (2016). Protein-protein interaction network of celiac disease. Gastroenterol Hepatol Bed Bench.

